# Supplementary material for: Pitfalls of Using NIR-Based Clinical Instruments to Test Eyes Implanted with Diffractive Intraocular Lenses
Source: Diagnostics (Basel). 2023 Mar 27;13(7):1259. doi: 10.3390/diagnostics13071259 (PMC10093131; doi:10.3390/diagnostics13071259)
Supplement: Supplementary file 1 [file diagnostics-13-01259-s001.zip › diagnostics-2219191-supplementary.pdf]

Supplementary Materials:

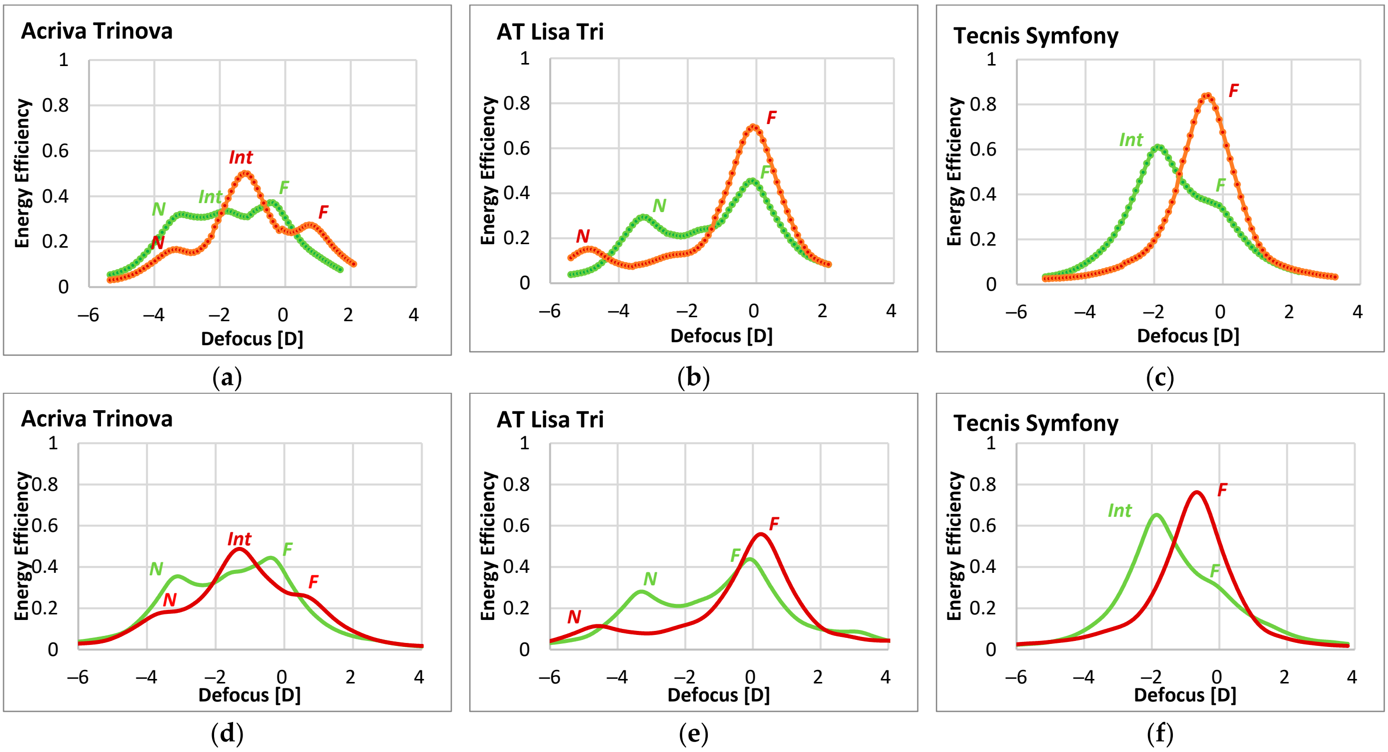

Figure S1. EE versus defocus (D) for the studied IOLs with 3.0mm IOL-pupil. Upper row (a), (b), (c): Experimental results (-●-●- green, -●-●- NIR). Lower row (d), (e), (f): simulated results (green and red solid lines represent green and NIR illuminations, respectively). Far (*F*), intermediate (*Int*) and near (*N*) foci are labeled for each lens and illumination.

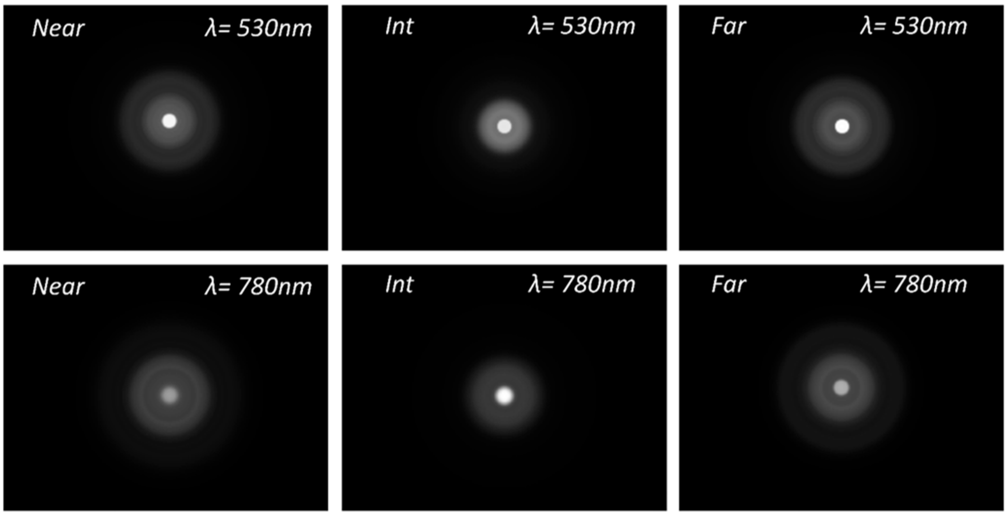

Figure S2. Simulated images of a pinhole test formed with Acriva Trinova IOL. Pupil 4.5mm. Gamma correction 0.45.

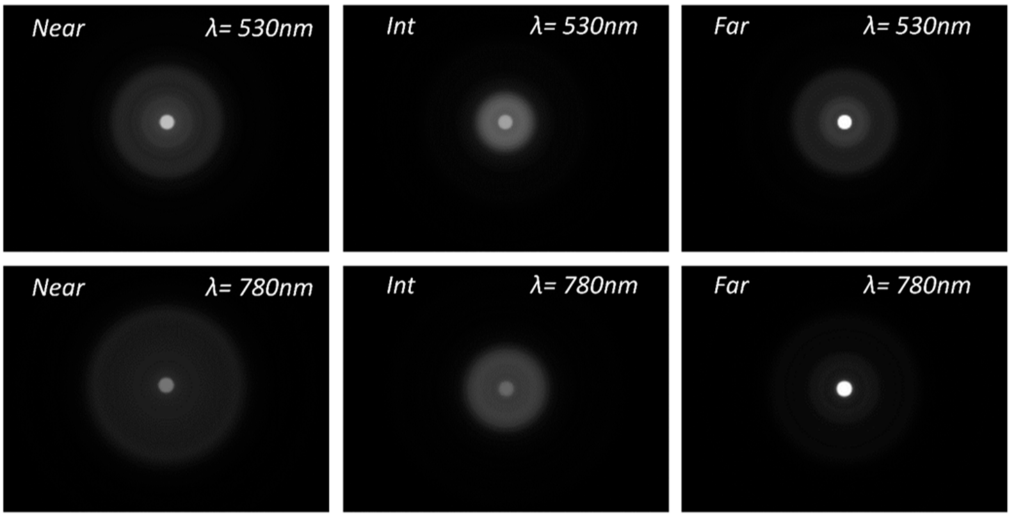

Figure S3. Simulated images of a pinhole test formed with AT Lisa Tri IOL. Pupil 4.5mm. Gamma correction 0.45.

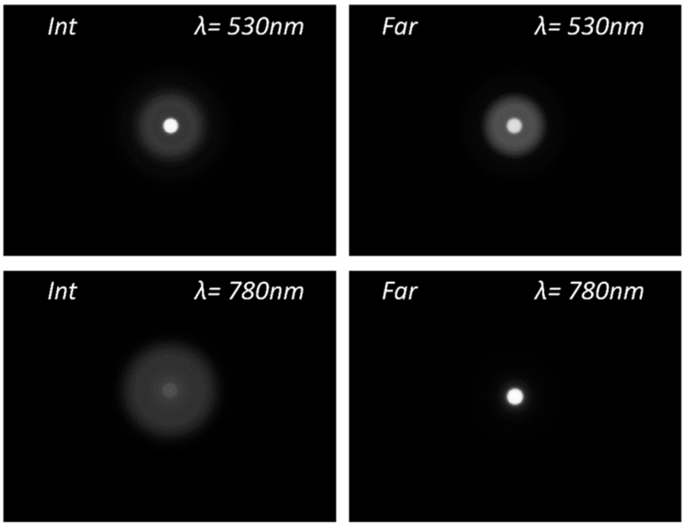

Figure S4. Simulated images of a pinhole test formed with Tecnis Symfony. Pupil 4.5mm. Gamma correction 0.45.
